# Cost‐effectiveness analysis of integrating screening and treatment of selected non‐communicable diseases into HIV/AIDS treatment in Uganda

**DOI:** 10.1002/jia2.25507

**Published:** 2020-06-19

**Authors:** David Sando, Alexander Kintu, Samson Okello, Peter Chris Kawungezi, David Guwatudde, Gerald Mutungi, Winnie Muyindike, Nicolas A Menzies, Goodarz Danaei, Stéphane Verguet

**Affiliations:** ^1^ Department of Global Health and Population Harvard T.H. Chan School of Public Health Boston MA USA; ^2^ Department of Internal Medicine Mbarara University of Science and Technology Mbarara Uganda; ^3^ Department of Community Health Mbarara University of Science and Technology Mbarara Uganda; ^4^ Department of Epidemiology and Biostatistics School of Public Health, College of Health Sciences, Makerere University Kampala Uganda; ^5^ Department of Non‐Communicable Diseases Prevention and Control Ministry of Health Kampala Uganda; ^6^ Department of Epidemiology Harvard T.H. Chan School of Public Health Boston MA USA

**Keywords:** HIV, antiretroviral therapy, non‐communicable diseases, hypertension, hypercholesterolaemia, diabetes, cardiovascular diseases, integration, sub‐Saharan Africa, Uganda

## Abstract

**Introduction:**

Despite growing enthusiasm for integrating treatment of non‐communicable diseases (NCDs) into human immunodeficiency virus (HIV) care and treatment services in sub‐Saharan Africa, there is little evidence on the potential health and financial consequences of such integration. We aim to study the cost‐effectiveness of basic NCD‐HIV integration in a Ugandan setting.

**Methods:**

We developed an epidemiologic‐cost model to analyze, from the provider perspective, the cost‐effectiveness of integrating hypertension, diabetes mellitus (DM) and high cholesterol screening and treatment for people living with HIV (PLWH) receiving antiretroviral therapy (ART) in Uganda. We utilized cardiovascular disease (CVD) risk estimations drawing from the previously established Globorisk model and systematic reviews; HIV and NCD risk factor prevalence from the World Health Organization’s STEPwise approach to Surveillance survey and global databases; and cost data from national drug price lists, expert consultation and the literature. Averted CVD cases and corresponding disability‐adjusted life years were estimated over 10 subsequent years along with incremental cost‐effectiveness of the integration.

**Results:**

Integrating services for hypertension, DM, and high cholesterol among ART patients in Uganda was associated with a mean decrease of the 10‐year risk of a CVD event: from 8.2 to 6.6% in older PLWH women (absolute risk reduction of 1.6%), and from 10.7 to 9.5% in older PLWH men (absolute risk reduction of 1.2%), respectively. Integration would yield estimated net costs between $1,400 and $3,250 per disability‐adjusted life year averted among older ART patients.

**Conclusions:**

Providing services for hypertension, DM and high cholesterol for Ugandan ART patients would reduce the overall CVD risk among these patients; it would amount to about 2.4% of national HIV/AIDS expenditure, and would present a cost‐effectiveness comparable to other standalone interventions to address NCDs in low‐ and middle‐income country settings.

## 1 Introduction

Non‐communicable diseases (NCDs) have become a major cause of disability and mortality among people living with HIV (PLWH) in sub‐Saharan Africa (SSA) [1]. This is largely due to rapidly increasing rates of risk factors, like hypertension, in PLWH [2, 3]. Previous cohort studies in SSA have shown that about 21% of PLWH were hypertensive, 22% had hypercholesterolaemia and 3% were diabetic [4, 5]. These high levels of risk factors increase the likelihood of cardiovascular diseases (CVD) such as stroke and ischaemic heart disease (IHD), jointly, and together with the likely direct effect of HIV infection on CVD outcomes [5, 6, 7, 8]. Concomitantly, improved viral suppression and life expectancy among PLWH stemming from expanded access to antiretroviral therapy (ART), now offer a greater time window for NCDs to develop to full manifestation as PLWH are ageing [9].

In recent years, there has been a dramatic rise in the incidence of CVD in the HIV‐infected population. For example previous studies have estimated a two‐fold increase in the 10‐year CVD risk among PLWH compared to HIV‐negative individuals [10, 11, 12, 13]. This can be attributed to the complex interplay of the inflammatory effect of HIV infection on the vascular walls, the increased prevalence of traditional risk factors such as hypertension, diabetes mellitus (DM), and hypercholesterolaemia, the adverse effects of some of highly active ART drug regimens, and the large disparities in access to timely screening and treatment of risk factors [6, 12, 14, 15, 16, 17, 18, 19]. In Uganda, where about 6% of the adult population lives with HIV [20], less than 7% of PLWH with hypertension have access to appropriate treatment [21]. Therefore, integration of routine screening and management of NCDs in HIV care and treatment settings offers an opportunity to curb the emerging NCD crisis that could otherwise jeopardize the health and economic benefits reaped through ART scale‐up. Indeed, integrating selected NCD services into ART delivery could leverage the past investments made towards HIV services to, additionally and effectively, deliver NCD treatment services and further reduce preventable deaths among PLWH by achieving economies of scope [22, 23].

In integrating screening and treatment of basic NCDs into ART delivery in SSA, the little evidence available so far supports the selection of appropriate NCD interventions to be added to current ART delivery practices. A few studies have examined the clinical benefits and cost implications of fully integrating screening, treatment and long‐term monitoring of risk factors like hypertension and hypercholesterolaemia among HIV‐infected patients [22, 23, 24]. A number of economic evaluations have also studied integrating NCD and HIV services; yet, they are often limited to screening and identifying basic risk factors, like hypertension, without covering the full cascade of NCD treatment (e.g. prevention of CVD) [25].

In this paper, we develop a cost‐epidemiologic model to study the health impact, costs and cost‐effectiveness of integrating basic screening and treatment services for hypertension, DM and hypercholesterolaemia in HIV treatment services in Uganda.

## 2 Methods

We examined the potential costs and health benefits associated with the integration of screening and treatment for hypertension, DM and hypercholesterolaemia, into HIV treatment services among PLWH receiving ART, compared to the current status quo (low coverage of NCD screening and treatment, see Table 1) in Uganda. We hypothetically evaluate outcomes (costs and health benefits) 10 years into the future (e.g. over 2017 to 2026) after NCD‐HIV integration start.

**Table 1:** Description of the interventions considered for the integration modality selected for screening and treatment of hypertension, hypercholesterolaemia, and diabetes mellitus (DM) within HIV treatment services in Uganda.

**Non‐communicable disease risk factor**	**Status quo**	**Integration modality: screen and treat PLWH receiving ART at the HIV clinic**
**Hypertension**	Few patients are screened for high BP	Full coverage of screening for BP
	Referral of individuals with high BP to hypertension clinic	Full coverage of treatment for individuals with high BP
**DM**	No patient is managed for DM	Screen and test patients for DM
	Refer patients with symptoms suggestive of DM to specialized clinic	Manage patients diagnosed with DM at the ART clinic
**Hypercholesterolaemia**	No screening for high total cholesterol	Full coverage of screening for high total cholesterol
	No treatment for patients with high total cholesterol	Full coverage of treatment for patients with high total cholesterol
Screening and treatment coverage	Hypertension (7%), DM (1%), hypercholesterolaemia (10%)	100% of PLWH receiving ART (64% of all PLWH in Uganda)

PLWH: people living with HIV. BP: blood pressure. Hypertension: systolic blood pressure >=140 mmHg and/or diastolic blood pressure >= 90 mmHg. Diabetes mellitus: blood glucose levels >=7.0 mm/L or >= 126 mg/dL. Hypercholesterolaemia: blood total cholesterol >= 5.0 mmol/L or >=190 mg/dL.

### 2.1 Intervention description

Compared with the status quo, integration would introduce treatment of PLWH receiving ART in public HIV clinics who were screened positive for NCDs. Public HIV clinics (within health facilities) are the designated point of care that offers HIV‐related services in Uganda. Such clinics provide non‐HIV services depending on the health facility level (e.g. in higher level facilities, another point of service for NCD care would exist). Most public clinics schedule services for specific conditions on particular days. For example a diabetic PLWH might not access services for diabetes during an ART pick up visit. She or he would instead have to return on a specific day of the week when the clinic offers diabetes services. The intervention – routine screening and treatment for hypertension, DM, and hypercholesterolaemia – would follow current national Ugandan guidelines [26]. The status quo would refer to the current health services environment where NCD treatment for PLWH is not delivered by HIV clinics.

**Figure 1:**
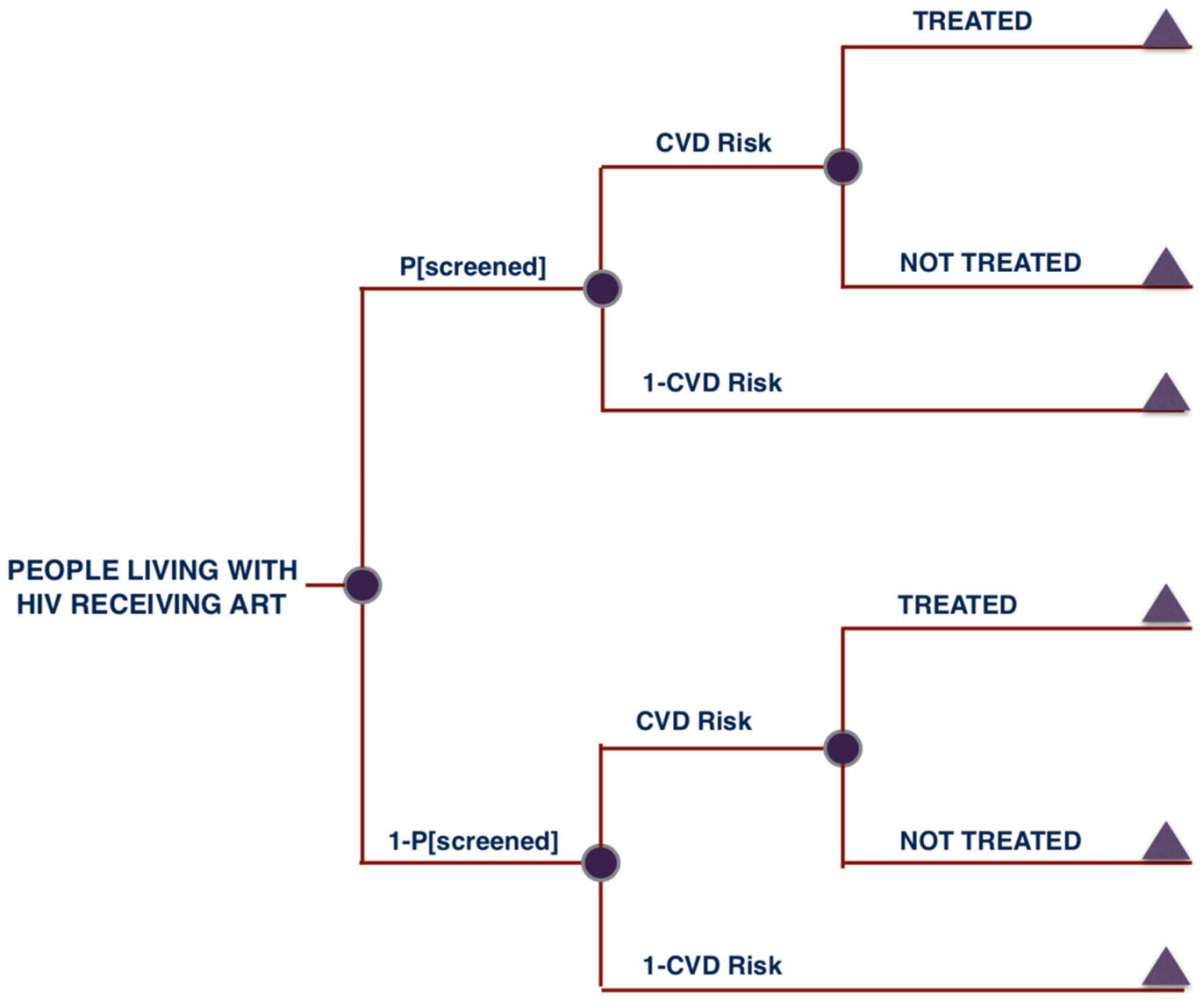
Simple decision tree describing the integration of cardiovascular disease (CVD) risk factor screening and treatment within HIV services in Uganda. The figure illustrates the differential cascade of care between patients (i.e. people living with HIV on ART) screened and those patients not screened for risk factors. CVD Risk: prevalence of CVD risk factors among people living with HIV receiving antiretroviral therapy (ART). P[screened]: percentage of people living with HIV receiving ART who are screened for hypertension, hypercholesterolaemia and diabetes mellitus. In the integration intervention scenario, P[screened] = 100% is assumed. Both branches of the decision‐tree model (screened vs. non‐screened patients) assume a constant state of non‐communicable diseases over the modelled time horizon (i.e. 10 years from integration start).

According to the national guidelines [26], hypertension is defined as systolic blood pressure (SBP) ≥140 mmHg and/or diastolic blood pressure (DBP) ≥90 mmHg; DM is defined as having blood test results for fasting blood glucose ≥7.0 mmol/L or random blood sugar ≥11.1mmol/L; and high total cholesterol is defined as total cholesterol ≥5.5mmol/L. In our analysis, we studied the cost‐effectiveness of integrating screening and treatment for hypertension, DM, and hypercholesterolaemia, across sex and age groups of PLWH (Figure 1).

### 2.2 Estimating the health benefits of integration

We built on Globorisk, a mathematical model that estimates the 10‐year risk of CVD (stroke and IHD) for a given individual, based on age, sex and risk factors including SBP and DBP levels, DM status, smoking status and cholesterol level [27]. The Globorisk model was further augmented by Kintu and colleagues to assess the 10‐year CVD risk among PLWH in Uganda [28]. Values for the model covariates (e.g. SBP, DPB levels) were drawn from a number of studies [21, 29, 30], which were conducted in Uganda or similar settings to reflect actual distributions of the risk factors and rates in the population. We then could estimate the 10‐year CVD risk with and without integration intervention (Table 1). Age‐ and sex‐based prevalence of risk factors in the population were sourced from nationally representative surveys (i.e. that used a randomly selected sample countrywide) including the national survey on NCDs (World Health Organization’s STEPwise approach to Surveillance (STEPs) survey [21]). Statistics on HIV prevalence were obtained from Uganda’s population‐based HIV impact survey [20]. Further detail on the underlying Globorisk model developed is given in the Appendix S1 (section 1).

For the status quo, we assumed no change in the baseline distribution of risk factor profiles in the population [21], and hence of the subsequent 10‐year CVD risk as estimated by Globorisk (i.e. projected CVD rates over 10 subsequent years based on past trends). For the integration impact on CVD, we drew from the published literature [19, 31, 32] to determine the net average proportional reduction in 10‐year CVD risk, hence in the occurrence of both fatal and non‐fatal CVD events (IHD, stroke) [10, 11, 12, 13, 14, 15]. We used average treatment effects from the published literature [19, 31, 32] to assign changes in CVD risk per individual patient: treated individuals were assumed to all experience the same proportional reduction in CVD risk. For individuals with more than one risk factor, treatment effect was assumed to be multiplicative [19, 29, 33]. The subsequent 10‐year CVD risks were then computed to estimate the number of CVD events with and without integration. In sum, the current adult Ugandan population (PLWH and non‐PLWH) was followed over 10 subsequent years (e.g. 2017 to 2026), for which the cumulative CVD risks were estimated, in the case of integration intervention versus status quo.

### 2.3 Estimating the costs of integration

We quantified the resources that would be required under both status quo and integration scenarios, along with their associated costs and prices of commodities and supplies, drawing from a set of previously outlined methodological principles [50].

We calculated the costs of managing risk factors and CVD events using an ingredients‐based approach and took the provider perspective. We incorporated prices and costs of all resources required, importantly human resources, laboratory equipment, and drug costs. We conducted extensive consultations with local experts in Mbarara University Referral Hospital (clinicians and hospital managers), Makerere University (physicians and academicians), and the Ministry of Health (policymakers), to define the feasible modalities for integration under Uganda’s existing health system. In the status quo, we assumed PLWH would receive risk factor treatment at levels similar to the general population (based on STEPs survey estimates [21]): 7% of PLWH with hypertension, 1% of PLWH with DM and 10% of PLWH with high cholesterol would receive treatment, respectively. Under integration, all PLWH receiving ART would be screened for risk factors and treated accordingly. Health workers’ time devoted to accomplishing the additional tasks required (both screening and treatment tasks) were fully incorporated. Such human resources costs were calculated based on current government salary scales for civil servants [34]. We assumed each ART patient would be screened once, and patients identified with risk factors would then receive annual screenings and drug refills during routine ART visits. Time spent by each specific health cadre during patient visit was estimated through consultation with local experts; and average time spent by each cadre was multiplied to the mean hour wage rate (gross salary) per cadre to obtain mean human resources costs per visit. Imaging and diagnostic costs were based on recommended standard national guidelines for NCD management [26]. Through expert consultations, we determined the average cost for minimum laboratory investigations and imaging tests by taking the mean cost of services in specialized public facilities and primary health centres [26, 35]. Drug costs were based on dosages from Uganda’s 2017 to 2018 Medical Store Department price catalogue [36].

Cost of managing CVD events was also based on standard national guidelines. We computed average cost of laboratory investigations, imaging tests, drugs and hospitalization per CVD event. A non‐fatal CVD event would receive additional drugs (as appropriate) and be clinically monitored annually. Monitoring of non‐fatal CVD events would include laboratory and imaging tests. We also computed cost of ART for the averted fatal CVD cases drawing from recently published estimates [37]. We focused on ART costs for people with an averted fatal CVD case because averted non‐fatal cases would still use ART without integration (in the status quo).

Lastly, for simplicity, our analysis excluded capital costs associated with infrastructure, buildings and other related facilities, under the assumption that those costs would remain covered by the already existing ART delivery services. All costs were estimated based on delivery by the Ugandan health system, and were discounted at 3% per year [40]. All prices were computed in local currency (UGS) and converted to 2017 USD using a mean exchange rate of USD 1 = UGS 3500 [38]. Table 2 provides a detailed description of all data inputs and corresponding sources; and additional information is provided in the Appendix S1 (section 2).

**Table 2:** Parameter inputs and corresponding sources used in assessing the cost‐effectiveness of HIV‐NCD integration services in Uganda. Indicated in parentheses are uncertainty ranges.

**Parameter**	**Estimate**	**Source**
**Demography**		
Uganda population	38,607,200	United Nations [46]
**HIV disease**		
Prevalence among 15‐49 year‐olds (%)	6.0 (5.5‐6.4)	HIV impact survey (2017) [20]
Proportion (%) of HIV‐infected individuals enrolled in ART programs	64	Global AIDS update [47]
**NCD risk factors**		
Prevalence of hypertension (%)		STEPs survey (2014) [21] and Appendix S1 (section 1)
30‐44 years	Men (24.5), Women (22.7),	
45‐59 years	Men (32.9), Women (41.5),	
60‐69 years	Men (35.3), Women (49.6)	
Prevalence of diabetes mellitus (%)		STEPs survey (2014) [21] and Appendix S1 (section 1)
30‐44 years	Men (2.7), Women (1.9)	
45‐59 years	Men (3.1), Women (4.2)	
60‐69 years	Men (4.0) Women (5.8)	
Prevalence of high cholesterol levels (%)		STEPs survey (2014) [21] and Appendix S1 (section 1)
30‐44 years	Men (5.1), Women (10.1)	
45‐59 years	Men (8.3), Women (14.8)	
60‐69 years	Men (7.3) Women (19.1)	
**Coverage of NCD risk factor treatment**		
Proportion currently on medication for hypertension (%)	7 (4 to 9)	STEPs survey (2014) [21]
Proportion currently on medication for diabetes mellitus (%)	1 (0 to 1)	
Proportion currently on medication for high cholesterol (%)	10 (6 to 14)	
**Treatment efficacy for NCD risk factor**		
Relative risk of stroke for hypertensive patients using single antihypertensive drugs	0.8 (0.7 to 0.9)	Turnbull et al. [32]
Relative risk of coronary events among diabetic patients with glycemic control	0.6 (0.6 to 0.7)	Stratton et al. [19]
Relative risk of coronary events among high cholesterol patients started on statin	0.7 (0.6 to 0.7)	LaRosa et al. [31]
**Costs** * (2017 USD, per specific type of care, per patient per year**)**		
**Hypertension**	$76 (32 to 144)	MoH Uganda [36]
Medical consultation	$2 (1 to 4)	
Laboratory and imaging tests (screening costs)	$33 (6 to 46)	
Medicines	$38 (24 to 90)	
**Diabetes mellitus**	$84 (22 to 143)	
Medical consultation	$2 (1 to 4)	
Laboratory and imaging tests (screening costs)	$5 (3 to 18)	
Medicines	$70 (17 to 109)	
**High cholesterol**	$97 (39 to 147)	
Medical consultation	$2 (1 to 4)	
Laboratory and imaging tests (screening costs)	$14 (7 to 20)	
Medicines	$79 (30 to 120)	
**Fatal CVD event** (< 30‐day survival)	$610 (420 to 1220)	
Average cost of hospitalization	$100 (50 to 150)	
Treatment cost	$510 (370 to 1070)	
**Non‐Fatal CVD event** (> 30‐day survival)	$810 (494 to 1560)	
Average cost of hospitalization	$100 (50 to 150)	
Treatment cost during the acute phase	$510 (370 to 1070)	
Annual treatment cost for non‐fatal CVD event	$200 (74 to 340)	
Cost of antiretroviral drugs	$265	Kimaro et al. (2017) [37]

ART = antiretroviral therapy; NCD = non‐communicable disease; CVD = cardiovascular disease.

public facilities. These costs reflect the current Ugandan national guidelines for standards of care.

a Costs are based on Uganda’s Ministry of Health (MoH) data and actual market prices for specific services in

### 2.4 CVD outcomes and cost‐effectiveness of integration

As described above, 10‐year CVD risks were obtained from Globorisk [27] and used to compute the number of CVD events (both fatal and non‐fatal events), in the integration and status quo scenarios, respectively. Each CVD event was then converted into Years of Life Lost (YLL) due to premature death and Years Lived with Disability (YLD) by applying disability weights from the 2013 Global Burden of Disease study [39]. We used Ugandan life tables [46] and age‐specific life expectancies to estimate YLLs associated with a premature CVD‐related death and YLDs associated with a non‐fatal CVD case, per five‐year age group (e.g. 50 to 55 year‐olds). CVD‐risks were estimated over 10 years into the future; hence, we assumed that CVD events would occur at mid‐time period (i.e. year 5 into the future), and we estimated YLLs and YLDs for each five‐year age group with respect to life expectancy for that age group forwarded five years into the future. Summing up YLLs and YLDs yielded disability‐adjusted life years (DALYs) which were discounted at 3% per year, and the total number of DALYs corresponding to each scenario (integration vs. status quo).

Per scenario, we computed the total costs as the sum of the costs of treating individuals with hypertension, DM, and hypercholesterolaemia; and the costs of treating CVD cases (both fatal and non‐fatal cases), and the ART costs. Incremental costs for integration were derived as the difference in total costs compared with status quo. We could then derive the net cost per DALY averted by integration.

### 2.5 Sensitivity analyses

We pursued four univariate sensitivity analyses, where we varied one input parameter at a time independently while maintaining values for the other input parameters unchanged. First, we varied the costs to consider how changes in input prices might impact our findings and to allow for uncertainty behind our costing approach for NCD screening and treatment. Therefore, we tested higher and lower cost values for treating NCDs and CVDs to capture different levels of health system provision. The lower cost would represent a scenario where care is provided within primary care facilities with limited human resources and absence of chest X‐rays, echocardiograms and electrocardiograms. The higher cost would represent a scenario where care is provided in specialized clinics with all recommended standards of care. Specifically, for treating hypertension, DM, and high cholesterol, the lower treatment cost was $32, 22 and 39 respectively; and the higher cost $144, 143 and 147 respectively. For treating CVD, the lower costs were $420 and 494 for fatal and non‐fatal events, respectively; and the higher costs $1220 and 1560. Second, for the treatment effects, we used confidence intervals from the published literature [19, 31, 32] to examine scenarios where integration would have either minimal or maximal impact. Specifically, for the effect size of treatment for hypertension, DM, and high cholesterol, the higher estimates of effectiveness had relative risks of 0.70, 0.59 and 0.60 respectively; and the lower estimates relative risks of 0.91, 0.72 and 0.74. Third, we also studied possible reduced coverage, which would correspond to the situation of NCD care coverage being lower in specialized HIV clinics: we assumed that integration would only reach 75% of PLWH receiving ART (instead of 100% in the base case).

### 2.6 Ethics approval

The study was approved by the ethical review boards of Mbarara University of Science and Technology (protocol number: 14/09‐17), the Uganda National Council of Science and Technology, and the Harvard T.H. Chan School of Public Health (protocol number: IRB16‐2062).

## 3 Results

**Figure 2:**
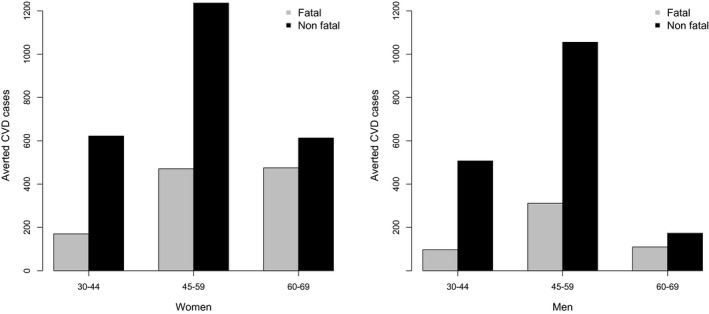
Averted cardiovascular disease (CVD) events (fatal and non‐fatal events), over 10 years, among people living with HIV receiving antiretroviral therapy in Uganda, per age group (30 to 44, 45 to 59 and 60 to 69 year‐olds) and sex, after integration.

Model outcomes were estimated for the current adult Ugandan population over 10 years of follow‐up. Examining the 10‐year CVD risk per age group and sex, we estimated a mean risk of 8.2% in 45‐69 year‐old PLWH women compared with 10.7% in 45‐69 year‐old PLWH men, in the status quo. This risk would decrease to 6.6% in women compared with 9.5% in men after integration (Appendix S1, Table S1). Consequently, with integration, the 10‐year absolute CVD risk among 45 to 59 and 60 to 69 year‐old women would decline by 19.6% (compared with the baseline absolute CVD risk) in overall CVD cases (corresponding to 1,705 and 1,090 cases, respectively). Comparatively, men would experience a 10.6% reduction (compared with baseline absolute CVD risk) in CVD cases among 45 to 59 and 60 to 69 year‐olds (1,365 and 285 cases, respectively) (Figure 2). Integration of risk factor treatment would reach about 255,000 PLWH aged 30 to 69 years who are currently enrolled in ART programmes. It would prevent an estimated 5,840 CVD cases (14% of all CVD cases) from occurring in PLWH within the 10 years after integration.

Table 3 summarizes the number of CVD cases that would be averted, the costs of providing NCD screening and treatment, the cost savings due to averted CVD cases and the net cost per DALY averted, per age group and sex, with integration. Integration of services for hypertension, DM, and hypercholesterolaemia would have an incremental cost‐effectiveness ranging from $8,800 to $1,400 per DALY averted, depending on the age and sex category. Targeting older age groups with higher CVD risks (e.g. 45 to 59 and 60 to 69 year‐olds), among both women and men, would be more cost‐effective. Overall, the incremental cost‐effectiveness ratios are high when compared with common thresholds of $500 to 1000 per DALY (about one half and one time Uganda’s gross domestic product (GDP) per capita).

**Table 3:** Averted cardiovascular disease (CVD) events, cost and net cost, and cost‐effectiveness of NCD‐HIV integration within HIV services in Uganda, disaggregated per age group (30‐44, 45‐59, 60‐69 year‐olds), and sex.

**Age** **group** (years)	**Cost of** **adding NCD** **services**	**Additional** **ART Cost**	**Cost** **savings due to averted CVD Cases**	**Net cost**	**CVD cases averted**	**Deaths** **averted**	**DALYs averted**	**Net cost** **per DALY averted**
**Women**								
**30‐44**	$65,544,000	$887,000	$2,985,000	$63,446,000	790	170	7,210	8,800
**45‐59**	$46,465,000	$1,934,000	$4,975,000	$43,424,000	1,705	470	13,350	3,255
**60‐69**	$11,206,000	$1,375,000	$2,039,000	$10,541,000	1,090	475	7,305	1,445
**Men**								
**30‐44**	$27,849,000	$470,000	$2,273,000	$26,046,000	605	95	4,705	5,535
**45‐59**	$21,431,000	$1,240,000	$4,105,000	$18,566,000	1,365	310	9,700	1,915
**60‐69**	$2,626,000	$301,000	$541,000	$2,386,000	285	110	1,700	1,400

Note:

all costs are expressed in 2017 USD. DALY = disability‐adjusted life year.

### 3.1 Sensitivity analyses

Table 4 displays the impact of the univariate sensitivity analyses on the cost‐effectiveness estimates (in net cost per DALY averted). Over the varying range of input parameters tested, the major changes observed followed the use of lower costs for treating hypertension, DM, and high cholesterol: for example the net cost per DALY averted in 60 to 69 year‐olds would then decrease to $540 and $505 in women and men, respectively. Likewise, cost‐effectiveness would be enhanced (net cost per DALY averted decreased) with higher treatment effectiveness estimates, and with higher cost estimates for CVD treatment.

**Table 4:** Results from selected univariate sensitivity analyses on costs, treatment effects, and coverage inputs for NCD‐HIV integration in Uganda among people living with HIV receiving antiretroviral therapy, disaggregated per age group (30‐44, 45‐59, 60‐69 year‐olds), and sex.

	**Age group** (years)	**Cost of adding NCD services**	**Additional cost of ART**	**Cost savings due to averted CVD events**	**Net cost**	**DALYs averted**	**Net cost per DALY averted**
	**Lower estimates for cost of treating hypertension, diabetes mellitus, and high cholesterol**						
Women	30‐44	27,901,000	887,000	2,985,000	25,803,000	7210	3580
	45‐59	19,312,000	1,934,000	4,975,000	16,271,000	13350	1220
	60‐69	4,614,000	1,375,000	2,039,000	3,949,000	7305	540
Men	30‐44	11,842,000	470,000	2,273,000	10,040,000	4705	2135
	45‐59	9,023,000	1,240,000	4,105,000	6,158,000	9700	635
	60‐69	1,098,000	301,000	541,000	858,000	1700	505
	**Higher estimates for cost of treating hypertension, diabetes mellitus, and high cholesterol**						
Women	30‐44	113,590,000	887,000	2,985,000	111,492,000	7210	15460
	45‐59	81,713,000	1,934,000	4,975,000	78,672,000	13350	5895
	60‐69	19,666,000	1,375,000	2,039,000	19,001,000	7305	2600
Men	30‐44	49,520,000	470,000	2,273,000	47,718,000	4705	10140
	45‐59	38,066,000	1,240,000	4,105,000	35,202,000	9700	3630
	60‐69	4,691,000	301,000	541,000	4,451,000	1700	2615
	**Lower estimate for cost of treating cardiovascular disease**						
Women	30‐44	65,544,000	887,000	1,241,000	65,190,000	7210	9040
	45‐59	46,465,000	1,934,000	2,136,000	46,264,000	13350	3465
	60‐69	11,206,000	1,375,000	942,000	11,638,000	7305	1595
Men	30‐44	27,849,000	470,000	945,000	27,374,000	4705	5815
	45‐59	21,431,000	1,240,000	1,755,000	20,917,000	9700	2155
	60‐69	2,626,000	301,000	249,000	2,678,000	1700	1575
	**Higher estimate for cost of treating cardiovascular disease**						
Women	30‐44	65,544,000	887,000	5,203,000	61,228,000	7210	8490
	45‐59	46,465,000	1,934,000	8,736,000	39,664,000	13350	2970
	60‐69	11,206,000	1,375,000	3,644,000	8,937,000	7305	1225
Men	30‐44	27,849,000	470,000	3,962,000	24,357,000	4705	5175
	45‐59	21,431,000	1,240,000	7,200,000	15,471,000	9700	1595
	60‐69	2,626,000	301,000	966,000	1,961,000	1700	1150
	**Higher estimates for NCD risk factor treatment effectiveness**						
Women	30‐44	65,544,000	1,751,000	5,961,000	61,333,000	14325	4280
	45‐59	46,465,000	3,223,000	8,598,000	41,091,000	22640	1815
	60‐69	11,206,000	2,048,000	3,149,000	10,104,000	11015	915
Men	30‐44	27,849,000	1,190,000	5,860,000	23,179,000	12055	1925
	45‐59	21,431,000	2,863,000	10,178,000	14,116,000	23290	605
	60‐69	2,626,000	629,000	1,224,000	2,031,000	3660	555
	**Lower estimates for NCD risk factor treatment effectiveness**						
Women	30‐44	65,544,000	564,000	1,872,000	64,236,000	4550	14120
	45‐59	46,465,000	1,064,000	2,531,000	44,998,000	7080	6355
	60‐69	11,206,000	568,000	709,000	11,065,000	2860	3865
Men	30‐44	27,849,000	114,000	499,000	27,464,000	1075	25580
	45‐59	21,431,000	672,000	1,981,000	20,122,000	4945	4065
	60‐69	2,626,000	181,000	290,000	2,517,000	980	2560
	**NCD risk factor treatment coverage reduced to 75%**						
Women	30‐44	47,848,000	665,000	2,239,000	46,275,000	5410	8555
	45‐59	33,937,000	1,450,000	3,732,000	31,656,000	10010	3160
	60‐69	8,148,000	1,031,000	1,529,000	7,650,000	5480	1395
Men	30‐44	20,382,000	353,000	1,705,000	19,031,000	3530	5390
	45‐59	15,669,000	930,000	3,079,000	13,520,000	7275	1860
	60‐69	1,921,000	226,000	406,000	1,741,000	1275	1365

Note:

all costs are expressed in 2017 USD. NCD = non‐communicable disease. CVD = cardiovascular disease (stroke and ischemic heart disease). DALY = disability‐adjusted life year.

## 4 Discussion

We pursued a cost‐effectiveness analysis of integrating NCD treatment among PLWH receiving ART in Uganda: we quantified the potential health gains, costs and cost‐effectiveness of integrating screening and treatment for hypertension, DM and high cholesterol in HIV services in Uganda. We developed a cost‐epidemiological model drawing from nationally representative surveys, the published literature, and Ugandan national guidelines. Our analysis is one of a few studies to date that have proposed an economic evaluation of integrating NCD care into HIV services in a low‐ and middle‐income sub‐Saharan African country [23, 24].

We found that NCD integration into existing ART clinics in Uganda would be associated with a decrease in the 10‐year CVD risk in PLWH. This is consistent with previous findings on the impact of NCD treatment on CVD incidence: for example, Ortegon and colleagues pointed that treating risk factors was associated with a decline in individual CVD risk directly proportional to the baseline risk level [41]. We also estimated a wide range in cost‐effectiveness, improving with targeting older age groups, from about $1,400 to 8,800 per DALY averted. The most cost‐effective scenario (around $1,400 per DALY averted) would correspond to targeting the oldest age group (60 to 69 year‐olds). Yet, given that life expectancy at birth is about 63 years and that the 60 to 69 year‐olds living with HIV represent a small population in Uganda [46], targeting all adults beyond age 45 would seem more appropriate. Our estimate ranges are comparable with previous cost‐effectiveness findings for low‐ and middle‐income countries (LMICs) recently reported by the *Disease Control Priorities* third edition, where Horton and colleagues [42] ranked different health interventions for LMIC settings showing large variations in net cost per DALY averted. For example, prevention of mother to child transmission of HIV and secondary treatment of CVD would cost $100 to $1000 per DALY averted; whereas, strategies for primary prevention of CVD would cost $1000 to $10,000 per DALY [42]. Our findings fall within the higher range of $500 to 1000 per DALY thresholds (around one half or one time Uganda’s GDP per capita) commonly reported [42]; and our estimates for younger age groups (e.g. 30 to 44 year‐olds) exceed such thresholds. In sum, in our modelling, the impact of NCD screening and treatment among PLWH would yield a modest CVD risk reduction (<1% in absolute risk reduction) at very high costs.

A series of articles have reviewed five potential modalities for NCD‐HIV integration in LMICs [7, 22, 23, 25]. Among them, only two modalities embraced a comprehensive care approach for NCDs, and emphasized providing combined screening and treatment services at the same delivery point. These two modalities would leverage on the existing HIV infrastructure to incorporate NCD services and to convert HIV clinics to serve patients with other chronic diseases who are not necessarily HIV‐positive. The already established strong health system for HIV services would have capacity to be augmented towards a “chronic care model.” Considering the limited funding for NCDs and the fragmentation of health systems in many developing countries, it may be most practical to begin NCD services provision with patients currently engaged in ART before expanding to others outside the scope of HIV programmes [1, 22]. In 2016/17, Ugandan national health expenditure on HIV/AIDS amounted to US$692 million [48] (out of an estimated $1.7 billion of total health expenditure [49]). The NCD‐HIV integration proposed here would present net costs of about $16 million (when targeting all PLWH receiving ART above age 30), which corresponds to roughly 2.4% of HIV/AIDS expenditure and 1.0% of total health expenditure.

Findings from our analysis could be used as inputs to NCD‐HIV integration policy design in Uganda. However, additional evidence would be required to support policy change. First, targeting NCD care to a specific population subgroup raises fundamental ethical dilemmas. Integrating NCD care to HIV services would demand careful examination of fairness principles underlying the decision to potentially deny such care to those not infected with HIV yet suffering NCDs. However, health systems in SSA have experience in formulating policy under such dilemmas, as in the case of integrating cervical cancer screening into HIV services [43], where higher risk of cervical cancer among HIV‐infected women was among several motivations [44, 45]. Second, leveraging on the already existing HIV infrastructure to introduce NCD care is likely to be less costly and has the potential to build health system capacity to address the growing NCD epidemic in the general population. Yet, further evidence on the impact of integration on patient waiting time, retention and potentially overburdening health workforce would be necessary.

Our analysis presents a number of important limitations. First, we limited our outcomes to health gains and a provider perspective: thus, we excluded non‐health benefits such as increased work productivity, and other indirect costs such as travel costs and time losses, which could be averted with prevention of CVD events. Second, we assumed similar treatment effectiveness and impacts would apply across all PLWH patients, and did not account for patient heterogeneity. We also used effectiveness estimates from studies conducted in high‐income countries, due to lack of data available in LMICs, whereas sensitivity analyses with additional treatment effectiveness estimates [30] and drug adherence considerations could be conducted. Likewise, supply chain systems that are weak in LMICs like Uganda and that can lead to delays and stockouts in drug delivery would likely diminish integration impact. In addition, due to lack of data, the model did not account for changes in other conditions which could be prevented by controlling NCDs such as retinopathy, renal diseases, and amputations; neither did we incorporate long‐standing NCDs with morbidity among older individuals, which could well reduce the health benefits (e.g. healthy life years) among the targeted older age groups. Another limitation pertains to the use of the Globorisk prediction model, which was developed using cohorts of non‐African populations [27]. Although we could not validate Globorisk to our Ugandan population, we recalibrated it by updating age‐ and sex‐specific CVD rates and risk factor prevalence using the nationally representative STEPs survey [21, 28]. Third, our cost estimates might not be nationally representative for Uganda, and our costs for screening and treatment were assumed to remain constant over 10 years, without consideration of changes in technology and evolution in drug prices over time. We considered the full costs implied by the national guidelines of standards of care (e.g. use of X‐rays and electrocardiograms) which may not be currently implementable in Uganda. And, we did not account for impact of scale on the cost of delivery (e.g. marginal cost decreasing with increasing volume of patients). However, to test the impact of our assumptions, we conducted a number of univariate sensitivity analyses (Table 4). Fourth, we did not consider NCD care provision among private facilities. Yet, public facilities account for the large majority of facilities delivering ART in Uganda, and NCD‐HIV integration via private facilities is largely left for future work. Also, adding risk factor treatment could be associated with longer clinical visits and waiting times, demanding additional capital costs to expand facilities. We however did not incorporate the possibility for longer waiting times associated with increased time taken to jointly provide HIV/NCD care, which could raise the additional capital costs needed to expand facilities and require more health workforce, especially in the context of already financially constrained HIV delivery systems. Lastly, for simplicity, we made several assumptions on integration coverage: PLWH receiving ART would be screened and treated; and the proportion of enrolled PLWH would remain constant at 64% (e.g. ART coverage in Uganda) over 10 subsequent years, even though, based on the current 90‐90‐90 goal (test 90% of PLWH, initiate 90% of PLWH on ART and attain viral suppression to 90% of PLWH on ART), expansion of ART would be expected in the coming years in Uganda.

## 5 Conclusions

As a conclusion, this paper offers preliminary evidence on the cost‐effectiveness of integration of screening and treatment services for hypertension, DM and high cholesterol in PLWH in Uganda and SSA (Box 1). Such approach could potentially be replicated to other sub‐Saharan African countries with similar CVD risk profiles and treatment costs for NCDs, drawing from local NCD prevalence data which may contrast with Uganda. Such integration could improve survival of PLWH and decrease the burden of disease in SSA. Yet, further research on the ethics, other costs and sustainability of NCD integration and chronic care models will be required to support the conversion of current health systems in low‐income countries towards addressing the rapidly expanding NCD epidemic (Box 2).

Box 1 **Policy implications**

- Integrating screening and treatment of NCDs into HIV care and treatment in Uganda could yield important health and financial benefits.
- Integrating screening and treatment for hypertension, high cholesterol, and diabetes mellitus among older PLWH in Uganda would present value for money comparable to other proposed interventions to address NCDs in low‐ and middle‐income countries.

Box 2 **Research implications**

- Economic evaluation research should be conducted on assessing the value for money of screening schedules for common NCDs in PLWH versus the whole population; and on expanding HIV care and treatment delivery toward a chronic disease care clinic model.
- Health services research should be conducted on the economies of scope and economies of scale along with the system requirements including human resources implications when integrating NCD services within HIV care and treatment delivery in sub‐Saharan Africa.

## Competing interests

We declare no competing interests.

## Authors’ Contributions

SV conceived the study. DS built the model, analyzed data, prepared results and wrote the first draft of the manuscript. AK, DG, GM, SB, SO, WM and PCK provided data and advice for the analysis. NAM and GD reviewed and provided advice on the simulation methods. All authors contributed to writing and reviewing the manuscript. SV had final responsibility for submitting to publication.

## Supporting information


**Appendix S1.** Supplementary appendix.
**Table S1.** Estimated cumulative 10‐year cardiovascular disease (CVD) risk (expressed in percent) among people living with HIV in Uganda, per age group (30 to 44, 45 to 59, or 60 to 69 year‐olds) and sex, with either current status quo or with integrating non‐communicable disease risk factor treatment among people living with HIV receiving antiretroviral therapy.
**Table S2.** Detailed description of the cost inputs used in the cost‐effectiveness model of NCD‐HIV integration in Uganda.
