# Supplementary material for: Cost‐effectiveness analysis of integrating screening and treatment of selected non‐communicable diseases into HIV/AIDS treatment in Uganda
Source: J Int AIDS Soc. 2020 Jun 19;23(Suppl 1):e25507. doi: 10.1002/jia2.25507 (PMC7305460; doi:10.1002/jia2.25507)
Supplement: Supplementary file 1 — Appendix S1. Supplementary appendix. Table S1. Estimated cumulative 10‐year cardiovascular disease (CVD) risk (expressed in percent) among people living with HIV in Uganda, per age group (30 to 44, 45 to 59, or 60 to 69 year‐olds) and sex, with either current status quo or with integrating non‐communicable disease risk factor treatment among people living with HIV receiving antiretroviral therapy. Table S2. Detailed description of the cost inputs used in the cost‐effectiveness model of NCD‐HIV integration in Uganda. [file JIA2-23-e25507-s001.pdf]

Supplementary appendix

Cost-effectiveness analysis of integrating screening and treatment of selected non-communicable disease risk factors into HIV/AIDS treatment in Uganda

by

David Sando, Alexander Kintu, Samson Okello, Peter Kawungezi, David Guwatudde, Gerald Mutungi, Winnie Muyindike, Nicolas A. Menzies, Goodarz Danaei, and Stéphane Verguet

1. Methods used to create a nationally representative dataset with individual-level data on CVD risk factors, HIV and ART status

We reproduce in this supplementary section a summary of the approaches described in great detail in Kintu and colleagues [1].

We started with the nationally representative 2014 Uganda STEPs survey [2,3] as the primary source of data on cardiovascular disease (CVD) risk factors. Second, we imputed HIV status (from the 2016 Uganda population-based HIV impact survey [4]) and antiretroviral therapy (ART) coverage (from Ugandan Ministry of Health data [5]). Third, we sourced documented differences in risk factor levels by HIV and ART status from a meta-analysis of sub-Saharan African country studies [6] to adjust the mean CVD risk factors, and subsequently we used the means and standard deviations from the STEPs survey to assign individual-level CVD risk factor values for the whole Ugandan population.

The risk equation model that underlied the CVD prediction model largely built on the previously established Globorisk prediction model [7], which can be calibrated for use in

different settings. For Uganda, the calibration was done by using average CVD risk factor levels in the whole population (i.e. from the STEPs survey) and fatal and non-fatal CVD rates for Uganda (i.e. from World Health Organization estimates).

The risk equation model was further modified as follows to take into consideration the direct effect of HIV on CVD [1]:

$$\gamma_i(t) = \gamma_{0,s}(t)\, exp[\, \sum_{r=1}^{4} \alpha_r\,(X_{r,i} - \bar{X}_{r,s,t}) + \sum_{r=1}^{4} \beta_r t\,(X_{r,i} - \bar{X}_{r,s,t}) + \sum_{r=1}^{2} \varphi_r sex_i\,(X_{r,i} - \bar{X}_{r,s,t}) + \delta\left(H_i - \bar{H}_{s,t}\right)]\quad , \qquad \text{(S.1)}$$

where $\gamma_i(t)$ is the CVD rate for individual i of HIV status H_i at age t; $\gamma_{0,s}(t)$ is the average CVD rate; r are CVD risk factors in the model; α_r is the hazard ratio corresponding to each risk factor; $\bar{X}_{r,s,t}'s$ are mean levels of risk factor r for each age-sex and HIV status; β_r is an interaction term between age and risk factor r, and φ_r captures the interaction between diabetes/smoking and sex; δ represents the direct effect of HIV on CVD; and $\bar{H}_{s,t}$ is the age-sex-specific prevalence of HIV.

We repeat this estimation (from equation S.1) for the subsequent nine years while updating the age of individuals in the model, and we eventually can estimate a 10-year CVD risk as the complement of the cumulative survival for the entire period of follow-up (i.e. 10 years).

Table S1 provides estimated cumulative 10-year CVD risks among people living with HIV (PLWH) in Uganda, per age group and sex, with either current status quo or with integrating risk factor treatment among PLWH receiving ART.

Table S1: Estimated cumulative 10-year cardiovascular disease (CVD) risk (expressed in percent) among people living with HIV in Uganda, per age group (30-44, 45-59, or 60-69 year-olds) and sex, with either current status quo or with integrating non-communicable disease risk factor treatment among people living with HIV receiving antiretroviral therapy.

	**Status quo**		**Integration**	
	Risk (%)	95% CI	Risk	95% CI
**Women**				
30-44 year-olds	1.3	1.1 - 1.5	1.1	1.0 - 1.2
45-59 year-olds	4.4	4.1 - 4.7	4.1	4.0 - 4.2
60-69 year-olds	14.6	13.5 - 15.3	14.1	13.2 - 15.0
**Men**				
30-44 year-olds	2.3	2.2 - 2.4	2.1	1.9 - 2.3
45-59 year-olds	7.4	7.4 - 7.8	7.2	6.8 - 7.6
60-69 year-olds	14.9	14.0 - 15.8	14.1	13.7 - 14.6

CI = confidence interval.

2. Costing methods implemented

We calculated the total costs of managing non-communicable disease (NCD) risk factors and CVD events using an ingredients-based approach and taking the health system provider perspective. We incorporated the costs of human resources (for patient consultations), laboratory investigations, and drug prices. We estimated the average mix of human resources needs through consultations with local experts (clinicians, NCD program officers, and academicians) based on the minimum requirements for a clinic to be certified for delivery of joint NCD and HIV services.

However, due to the different levels of health facilities (e.g. dispensaries, health centers, district-level hospitals, referral hospitals) engaged with the provision of HIV services, we used such variations in facility levels to derive possible lower and higher cost estimates. For instance, HIV clinics in referral hospitals and specialized centers are often run by medical specialists and highly skilled nurses, while HIV clinics in lower level health facilities are usually attended by general medical doctors, clinical officers and nurses. The existing variations in the qualifications of staff in HIV clinics from different levels of health facilities will imply differences in human resources costs for the provision of care to HIV patients. We used the mix of human resources types from both health centers and district-level hospitals to derive an average cost corresponding to the human resources needs.

As a result, Table S2 below shows five types of health workers that would be required to provide basic HIV and NCD services, including medical officers, nursing officers, laboratory technologists, radiology technicians, and pharmacy assistants. Subsquently, we computed the additional time (to deliver NCD services) that would be required by each worker type through

expert interviews, and we could extrapolate the human resources cost per patient visit. We used the current Ugandan government salary scale (gross salary) to derive a cost per visit for each worker type [8].

According to Uganda's national guidelines on NCD management [9], hypertensive patients are recommended to undertake cardiothoracic examinations using X-rays, echocardiograms (ECHO) and electrocardiograms (ECG), annually. Therefore, we computed the costs corresponding to these investigations based on the current prices of these equipments in public settings, with input from local experts. The blood tests, namely full blood counts, are part of the national guidelines for the management of HIV patients. Based on expert opinions, we assumed that there would be six monitoring tests for diabetic patients per year using rapid blood glucose test; and we computed the cost for these tests based on Uganda's medical store price list [10]. Following the national guidelines, hypercholesterolemia patients would receive a blood test for cholesterol levels annually.

Drawing from the national guidelines [9], we also assumed each CVD case would receive computed tomography (CT) scan imaging services and a set of laboratory tests (e.g. full blood counts) during the first facility visit after a CVD event and a follow-up CT-scan within the first year. For higher estimates for CVD treatment, we assumed patients would receive magnetic resonance imaging (MRI) scan instead of CT scan. The total cost of managing a CVD case during the first clinic visit was estimated to be $510. A non-fatal CVD case was expected to continue with treatment and in each consecutive year would cost about $200 related to providing drugs and laboratory tests.

Table S2. Detailed description of the cost inputs used in the cost-effectiveness model of NCD-HIV integration in Uganda.

**Part A: Cost for human resources (per patient visit)**					
**Personnel**	**Time (hour)**	**Monthly wage**	**Average cost**	**Lower estimate**	**Upper estimate**
General medical officer	0.42	366.70	0.95	0.30	2.50
Nursing officer	0.25	253.08	0.40	0.12	0.62
Lab technologist	0.17	253.08	0.26	0.26	0.26
Radiology technologist	0.17	253.08	0.26	0.26	0.26
Pharmacy dispenser	0.17	253.08	0.26	0.26	0.26
**Subtotal**			**2.14**	**1.20**	**3.90**
**Part B: Cost of laboratory and imaging services (per patient-year)**					
**Disease**	**Frequency per year**	**Unit cost per test**	**Average cost**	**Lower estimate**	**Upper estimate**
**Hypertension**					
Chest X-ray	1	5.56	5.56		5.56
ECHO & ECG	1	27.78	27.78		27.78
Laboratory tests (covered by current ART services; one additional test per year based on the guidelines)		12.60	12.60		12.60
**Subtotal**			**33.34**		**45.94**
**Diabetes mellitus**					
Glucose tests (assumed 3 tests (vs. 6 tests) in lower level facilities)	6	0.83	4.98	2.49	
Other laboratory tests (CDC, RFT, LFT) (covered by current ART services; one additional test per year based on the guidelines)		13.10			13.10
**Subtotal**			**4.98**	**2.49**	**13.10**
**Hypercholesteromia**					
Lipid profile tests (done twice per year, for lower estimate, only once per year)	2	6.95	13.90	6.95	13.90
Other laboratory tests	1				5.60
**Subtotal**			**13.90**	**6.95**	**19.50**
**CVD events**					
CT scan (no change for lower/higher estimates)	2	138.89	277.78		
Laboratory tests (same range of tests as for hypertensive patients)	2	33.34	66.68	66.68	66.68
MRI (considered only for higher estimate in place of CT scan)	1				**400.00**

**Subtotal**			**344.46**	**66.68**	**466.68**
**<u>Part C</u>: Cost of drugs (per year)**					
Hypertension (Bendrofluazide)		38.00	38.00	38.00	38.00
Diabetes mellitus (Metformin)		70.00	70.00	70.00	70.00
Hypercholesterolemia (Statin)		79.00	79.00	79.00	79.00
**PART D: Total cost per disease (PART A + PART B + PART C)**					
	**Consultation cost (human resources costs)**		**Laboratory and imaging costs**	**Drugs**	**Total**
	**Cost per visit**	**Cost for all visits** *(HTN = 2 visits, DM = 4 visits, HCL = 2 visits)*			
Hypertension	2.14	4.28	33.34	38.00	**75.62**
Diabetes mellitus	2.14	8.56	4.98	70.00	**83.54**
Hypercholesterolemia	2.14	4.28	13.90	79.00	**97.18**
CVD event (consultation cost included in hospital admission)	2.14	42.8	344.46	123.00	**510.26**

All costs are expressed in 2017 USD.
Echo= echocardiogram; ECG = electrocardiogram; CDC = cell blood counts; RFT = renal function test; LFT = liver function test; CT scan = computer tomographic scan; MRI = magnetic resonance imaging; NCD = non-communicable disease; CVD = cardiovascular disease; DM = diabetes mellitus; HTN = hypertension; HCL = hypercholesterolemia.

References

1. Kintu A, Sando D, Guwatudde D, Bahendeka S, Kawungezi P, Mutungi G, Muyindike W, Menzies NA, Okello S, Danaei G, Verguet S. Quantifying the burden of cardiovascular diseases among people living with HIV in sub-Saharan Africa: findings from a modeling study for Uganda. Under review by Journal of the International AIDS Society.
2. World Health Organization. A framework for surveillance: The WHO STEPwise approach to Surveillance of noncommunicable diseases (STEPS). Geneva: World Health Organization, 2003.
3. Ministry of Health, Uganda. Non-Communicable Disease Risk Factor Baseline Survey. 2014. Available from: http://ghdx.healthdata.org/record/uganda-steps-noncommunicable-disease-risk-factors-survey-2014 (accessed March 1, 2020).
4. Ministry of Health, Uganda. Uganda Population-Based HIV Impact Survey 2016-2017. Available from: https://afro.who.int/sites/default/files/2017-08/UPHIA%20Uganda%20factsheet.pdf (accessed March 1, 2020).
5. Uganda AIDS Commission. Uganda HIV/AIDS country progress report July 2016-June 2017. Available from: https://www.unaids.org/sites/default/files/country/documents/UGA_2018_countryreport.pdf (accessed February 29, 2020).
6. Dillon DG, Gurdasani D, Riha J, Ekoru K, Asiki G, Mayanja BN, et al. Association of HIV and ART with cardiometabolic traits in sub-Saharan Africa: a systematic review and meta-analysis. International Journal of Epidemiology 2013; 42:1754-71.
7. Hajifathalian K, Ueda P, Lu Y, Woodward M, Ahmadvand A, Aguilar-Salinas CA, et al. A novel risk score to predict cardiovascular disease risk in national populations (Globorisk): a pooled analysis of prospective cohorts and health examination surveys. Lancet Diabetes Endocrinol 2015; 3:339-55.
8. Ministry of Public Finance, Uganda. Salary structure. Available from https://publicservice.go.ug/download/salary-structure-fy-2018-2019-schedule-1-12/ (accessed March 1, 2020).
9. Ministry of Health, Uganda. Uganda clinical guidelines 2016. National guidelines for management of common conditions. Available from: http://apps.who.int/medicinedocs/documents/s23532en/s23532en.pdf (accessed March 1, 2020).
10. National Medical Stores, Uganda. Essential medicines, essential health supplies, specialist medicines, specialist health supplies, basic equipment, October 2010. Available from: http://www.nms.go.ug/images/imported_files/CATALOG_OCT_2010.pdf (accessed March 1, 2020).
